# Effects of acute exposures of 2,4,6-trinitrotoluene and inorganic lead on the fecal microbiome of the green anole (*Anolis carolinensis*)

**DOI:** 10.1371/journal.pone.0208281

**Published:** 2018-12-06

**Authors:** Karl J. Indest, Steven J. Everman, James H. Lindsay, Carina M. Jung, Jared C. Smith, Sandra B. Newell

**Affiliations:** 1 The Environmental Laboratory, U.S. Army Engineer Research and Development Center, Vicksburg, Mississippi, United States of America; 2 Bennett Aerospace, Cary, North Carolina, United States of America; Nanjing University, CHINA

## Abstract

Microbiome studies focused on ecologically relevant vertebrate models like reptiles have been limited. Because of their relatively small home range, fast maturation, and high fecundity, lizards are an excellent reptilian terrestrial indicator species. For this study we used the green anole, *Anolis carolinensis*, to assess the impact of military relevant contaminants on fecal microbiome composition. Fourteen day sub-acute exposures were conducted via oral gavage with 2,4,6-Trinitrotoluene (TNT) and inorganic lead at doses of 60 mg/kg and 20 mg/kg of body weight, respectively. Body weights and food consumption were monitored and fecal samples were collected for high-throughput 16S rRNA gene amplicon sequencing and analytical chemistry at days 0 and 15. At the end of the study, liver and gut were harvested for body burden data. Chemical analysis confirmed accumulation of TNT, TNT transformation products, and lead in liver tissue and fecal samples. Bacterial community analysis of fecal material revealed significant differences between day 0 and day 15 of TNT exposed anoles with an operational taxonomic unit (OTU) within the genus *Erwinia* representing 32% of the microbial community in TNT exposed anoles. Predictable changes in gut microbiome composition could offer an easily assayed, noninvasive biomarker for specific chemical exposure providing enhanced scientific support to risk assessments on military installations.

## Introduction

Explosives and metals are common military contaminants that have impacted soil and groundwater at many U.S. Army installations resulting in significant contamination of large tracts of military lands [1, 2]. Due to rapid urbanization, these lands have become important refuges that support wildlife and these contaminants have the potential to negatively impact local/regional ecosystem health. Lizards are commonly found on U.S. Department of Defense (DoD) installations and as a result have been used to assess ecological risk of military relevant contaminants. The western fence lizard, *Sceloporus occidentalis*, and the green anole, *Anolis carolinensis* to a lesser extent, have been used to evaluate the toxicity of several toxicants including explosives and metals [3–5], pesticides [6, 7], and endocrine disrupters [8]. These lizard species are not herbivorous but rather feed on various invertebrates [9]. As a result, they make excellent contaminant indicator species because they are continuously sampling their environment, directly ingesting a contaminant or feeding on organisms that may have bioaccumulated a contaminant. In general, lizards are an excellent indicator species due to their relatively small home range, fast maturation, and high fecundity [9].

While mammalian microbiome-based studies have become common-place in the medical literature, there have been limited gut microbiome studies focused on ecologically relevant animal models like reptiles. In general, fundamental gut microbiome characterizations are lacking for lizards with only a few published studies comparing the gut microbiomes in relation to diet or adaptive radiation [10–16]. The gut microbiome, defined as the microbiota associated with the host species digestive tract, is often an initial point of contact with the environment for most host organisms. As a result of these interactions a number of studies indicate that the digestive tract microbiome can directly impact host exposure to some environmental chemicals [17, 18]. The influence of gut microbes on xenobiotic metabolism has historically focused on assessing the metabolic activity of gut microbial communities on antibiotics and botanicals [19, 20]. More recently, microbiome studies have demonstrated transformation of toxicants, most notably arsenic and PAHs, into potentially toxic metabolites [21–23]. These studies illustrate that the microbiome affects host exposure to toxicants in many ways such as increasing bioavailability and biotransformation. Studies have also demonstrated that contaminants like arsenic, which are transformed by the gut microbiome, in turn perturb gut microbiome composition and metabolism [24–27]. Recently, the utility of gut microbiome composition was investigated as a pre-clinical tool for identifying exposures to specific heavy metals using the rat animal model [28]. The authors identified bacteria with higher numbers of iron-importing gene orthologs in animals exposed to arsenic and nickel compared to exposures to cobalt, chromium, and cadmium.

As a result of these recent findings, we hypothesize that predictable changes in lizard gut microbiome composition in response to exposure to different classes of contaminants, may serve as an easily assayed, noninvasive biomarker for chemical exposure. Fundamental information regarding anole microbiome composition and stability, and the response of microbiome composition to contaminant exposure are required to achieve this objective. For this study, we leveraged existing toxicology data from previous studies to address impacts these compounds have on host fecal microbiome. For example TNT acute, subacute, and subchronic oral toxicity data is well established for the western fence lizard [3, 4] with a lowest-observed-adverse effect level of 25 mg/kg/d and a no-observed-adverse effect level of 15 mg/kg/d. In addition, significant sub-lethal effects of 10 and 20 mg Pb/kg/d was observed for adult and juvenile male lizards in sub chronic exposures [29]. Based on these studies we selected 60 and 20 mg/kg of body weight for TNT and lead, respectively.

## Materials and methods

### Experimental animals and husbandry

Wild-caught male *Anolis carolinensis* were purchased from Carolina Biological Supply and housed at Environmental Toxicology Research Facility at the US Army Engineer Research and Development Center (Vicksburg, MS). Each anole was housed in its own individual 42-L glass terrarium, complete with vertical plastic plant substrate. Terraria were placed on racks, with each animal having access to a shared 60-watt heat lamp and 24 W UVB bulb (one per two tanks). All terraria were misted daily (2X) and fecal matter, shed skin, and any uneaten portions of prey items (e.g., cricket legs) were removed from terraria daily. The room and individual heat lamps were on a 12:12 light:dark cycle, and the room temperature was held at 23 ± 2°C. Anoles were fed 3–4 crickets on Monday, Wednesday, and Friday and supplemented with multi-vitamins and calcium. Once acquired, anoles were acclimated in the laboratory for a minimum of two weeks prior to testing. Housing conditions and the experimental design adhered to pre-approved standards set by the U.S. Army Engineer Research and Development Center’s Environmental Laboratory Institutional Animal Care and Use Committee (IACUC). Activities were approved and monitored by this IACUC for both holding (EL-6009-2014-3/EL-6009-2018-1) and experimental (EL-6009-2016-6) protocols. The research was not conducted using sedated, dead, or inanimate surrogates, because only fully functioning lizards exhibit the reactions that determine the potential effects of exposure. The minimum number of lizards was used to provide for robust statistical interpretation of data.

### Exposures and specimen sampling

Fourteen-day sub-acute exposures were conducted with 2,4,6-trinitrotoluene (TNT) and lead nitrate (Sigma-Aldrich) at doses of 60 and 20 mg/kg of body weight, respectively. Military-grade TNT (containing ≤ 1% impurities) was obtained from the Holston Army Ammunition Plant (Kingsport, TN, USA). Both TNT and lead were mixed and stirred continuously until solubility was visually confirmed. Anoles were orally dosed daily using soybean oil as a carrier with controls receiving only soybean oil. During forced ingestion, or gavage, lizards could have been subjected to distress from the procedures. Anesthesia was not administered for potential interference with the uptake of the compounds, however only trained personnel were used for this approach, limiting the distress and potential for injury. Fresh fecal pellets (residence time of less than four hours in the terraria) were collected as available for microbial analysis during the two-week acclimation period and before, during, and after the 14 day exposure period (on day 15) as well. Additional fecal pellets were collected during the exposure period for TNT and lead analysis. Body weights were recorded at the beginning and end of the exposure period. Lizards were euthanized by means of cervical dislocation at the end of the experiment (day 15) including the dissection of liver and gut tissues. This is recognized as an acceptable form of euthanasia for reptiles according to the American Veterinary Medical Association’s Guidelines for the Euthanasia of Animals (https://www.avma.org/KB/Policies/Documents/euthanasia.pdf). Also, those responsible for performing cervical dislocation have been properly trained and consistently applied it humanely and effectively. Weight, snout-vent length, morphological characteristics, and tissue weights were recorded following sacrifice. These tissues were weighed, swabbed for intestinal fecal material collection for a final microbiome analysis, and stored at -20°C prior to chemical analysis.

### Chemical analysis

Tissue and fecal samples were homogenized via sonication in a water bath prior to analysis. TNT and TNT transformation products were measured by high performance liquid chromatography (HPLC) using an Agilent 1200 HPLC equipped with a Phenomenex (Synergi 4-μm Hydro-RP, 80A 250 x 4.6 mm) and a Restek (Pinacle II Biphenyl, 5 mm, 150 mm x 4.6 mm) reversed-phase column, employing a modified USEPA method 8330B (2006). Elemental analysis was performed by inductively coupled plasma atomic emission spectroscopy and mass spectrometry (Perkin Elmer Optima 3000 ICP-AES and Elan 6000 ICP-MS) using modified US EPA Methods 6010B (1996) and 6020 (1994).

### Fecal collection, DNA extraction, and high-throughput 16S rRNA gene amplicon sequencing

Fresh feces from anoles were collected by placing the deposited feces into a sterile centrifuge tube using a sterile inoculating loop or during dissections at the termination of the experiment. Samples were kept frozen at −20°C in the laboratory prior to DNA extraction. DNA extraction, high throughput sequencing, and sequence analysis was accomplished as described previously [30]. Briefly, as a semi-quantitative survey method we used 16S rRNA community analysis via next generation sequencing with the Illumina MiSeq platform (Illumina, Inc., San Diego, CA). DNA was extracted from fecal pellets via DNeasy PowerSoil Kit (Qiagen, Hilden, Germany) and resultant DNA was amplified with uniquely barcoded primers specifically designed for paired-end 16S rRNA gene amplicon sequencing with the Illumina MiSeq and encompassing approximately 300 bp; spanning the V4 region (515–806 bp) of the 16S rRNA gene [31]. Amplicons were combined and normalized to 20 pmol and further combined with 30% PhiX control according to Illumina MiSeq instructions. A 300 cycle MiSeq kit was employed for sequencing. Quality filtered qseq files were analyzed offline using QIIME [30] for supporting analysis such as demultiplexing and quality filtering, closed reference OTU picking, taxonomic assignment, phylogenetic reconstruction, alpha and beta diversity analyses, and visualizations. Samples were rarefied to 20k reads each and default parameters were used unless otherwise stated. For each sample, observed OTU and PD whole tree estimates were obtained by calculating the mean of 10 iterations of subsampling at 20k sequences. In those instances where significant differences were observed between overall microbial community composition at day 0 and day 15, abundances of Kyoto Encyclopedia of Genes and Genomes (KEGG) orthologous genes and pathways [32] were estimated using the PICRUSt tool (phylogenetic investigation of communities by reconstruction of unobserved states) with default parameters [33]. All sequencing data was deposited under SRA accession: PRJNA504817.

### Statistical analysis

Permutation multivariate analysis of variance [34] (PERMANOVA, adonis in R) was used to test for effects of acclimation period, source material, soybean oil, and TNT or lead on observed alpha and beta diversity metrics. R^2^ values reveal the approximate variation accounted for by each factor and p-values less than 0.05 were considered significant. To account for high variation among individuals, while tracking changes within each individual, permutations were also restricted to within individual anoles. As a result, the number of anoles used in each analysis varied based on the ability to acquire two samples per anole at critical times such as the beginning and end of the acclimation/acute studies. Effects on observed OTUs and PD whole tree diversity estimates were determined via PERMANOVA using Euclidean distance as the distance metric with up to 10k permutations. Input for each PERMANOVA was a numerical matrix with samples as rows and a single column with either mean Faith's Phylogenetic Diversity estimates (PD whole tree alpha diversity) or number of observed OTUs as the values. Effects on overall fecal microbial community and community composition at various taxonomic levels was determined via PERMANOVA using Weighted UniFrac Distance [35] as the distance metric with up to 10k permutations and LEfSe with default parameters (Kruskal Wallis test p <0.05 and LDA score >2) [36], respectively. KEGG orthologous genes and pathways that were significantly different between treatment groups were determined using LEfSe (linear discriminant analysis effect size) with default parameters (Kruskal Wallis test p <0.05 and LDA score >2) [36].

## Results

### Effects of acclimation on wild-caught anoles

Prior to exposing wild-caught male anoles to experimental conditions, reproducibility and stability of fecal microbiome composition signatures was assessed in newly collected, wild-caught male anoles and anoles acclimated to laboratory conditions for at least two weeks. Fecal samples were collected upon arrival (n = 20) and following the two week acclimation period (n = 20). During the acclimation period anoles were fed a consistent diet of crickets and provided a common supply of dechlorinated municipal water. As a result, it was anticipated that a reduction in overall microbial alpha diversity would be observed in the acclimated anoles compared to un-acclimated anoles. Similarly, it was further anticipated that microbial community composition as measured by beta diversity would be significantly different between the groups. Surprisingly, significant differences were not observed using either alpha diversity metric (S1 Fig) or overall microbial community composition (S2 Fig) in repeat sampled acclimated anoles compared to day of arrival. PERMANOVA revealed that very little of the variation between overall microbial community composition could be attributed to the effects of time (~4%) which in this case was the acclimation period. Most of the variation in microbial community composition (63%) was attributed to the individual anole, indicating that the fecal microbiome in our experimental system is intrinsically variable between individuals. Despite this variability, members of the Bacteroidaceae were consistently the predominant phylotype present in both the tested conditions (~51% before and ~60% after acclimation), followed by members of the Fusobacteriaceae (~8% before and ~11% after acclimation) and Enterobacteriaceae (~19% before and ~7% after acclimation) (S3 Fig).

### Effects of soybean oil and source material

Microbiome analysis was performed on control anoles dosed with soybean oil (n = 6) to exclude any possibility that soybean oil could be a factor in observed differences between anole fecal microbial communities at day 0 and day 15. No significant differences in either alpha diversity metric (data not shown) or overall microbial community composition (S4 Fig) were observed over the 14 day exposure period. PERMANOVA further revealed that only ~13% of the variation in overall microbial community composition between day 0 and 15 could be attributed to the effects of soybean oil. Additionally, an identical analysis was performed on the source material (fresh fecal pellets collected at day 0 vs dissected hindgut intestine contents at day 15) (n = 9) used for DNA extraction to exclude the possibility that this may have also had an effect on observed differences between the microbial communities. No significant differences in either alpha diversity metric (data not shown) or overall microbial community composition (S5 Fig) were observed over the 14 day exposure period. This result was consistent with a previous lizard fecal microbiome study that indicated that fecal samples are largely representative of the hindgut bacterial community [14]. Therefore, these results support the observation that our repeat sampling experimental design/methods of control anoles does not result in significant differences in overall microbial community composition during the 14 day exposure period regardless of soybean oil and source material.

### Subacute TNT exposure

**Fig 1:**
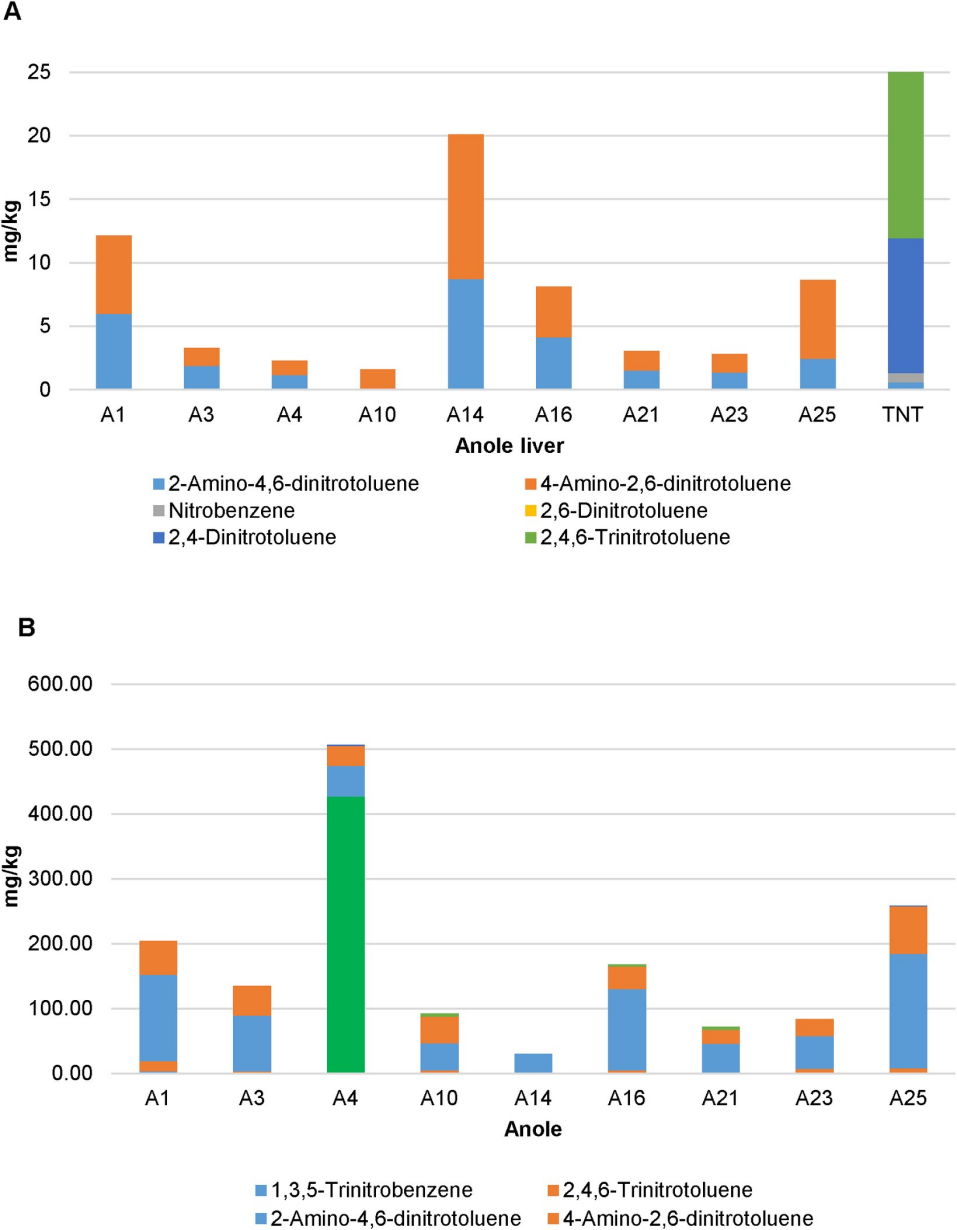
Analysis of TNT and TNT transformation products in liver (A) and fecal samples (B) collected from anoles exposed to TNT.

**Fig 2:**
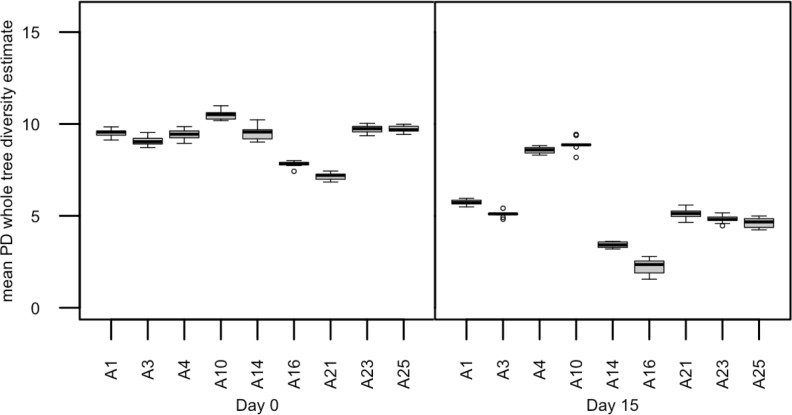
Comparisons of PD whole tree alpha diversity of TNT dosed male anoles’ fecal microbiome at day 0 and day 15.

**Fig 3:**
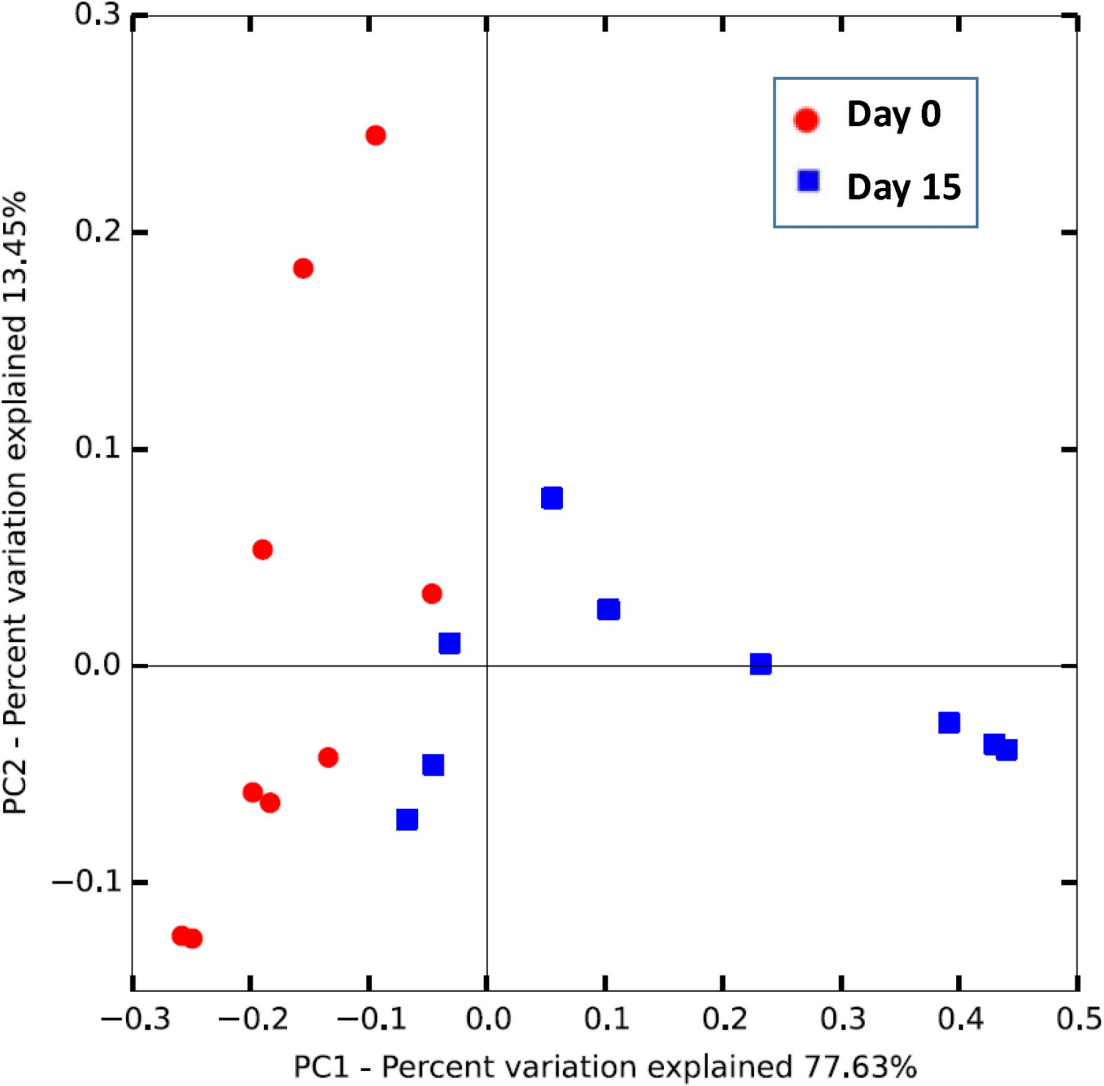
Principal coordinate analysis (PCoA) based on weighted UniFrac distance among day 0 and day 15 microbiome of anoles dosed with TNT.

Acclimated adult male anoles (n = 9) were subjected to daily sub-acute oral doses of TNT (60 mg/kg) in soybean oil for 14 days. Mean percent weight loss was 2.8% (±22.4%) over the course of the 14-day exposure period suggesting a limited but varied TNT response to body weight (data not shown). TNT and transformation products were detected both in anole liver tissues (Fig 1A) as well as in fecal samples (Fig 1B). The predominant transformation product detected in the feces was 2-amino-4,6-dinitrotoluene followed by 4-amino-4,6-dinitrotoluene. In contrast, in the liver 4-amino-4,6-dinitrotoluene was the predominant TNT transformation product followed by 2,4-dinitrotolulene, which was not detected in the feces. Microbiome analysis was conducted on TNT dosed anoles at day 0 and day 15. Significant differences were observed in mean PD whole tree (df = 1, pseudo-F = 36.455, p = 0.0005) (Fig 2) diversity estimates. Significant differences were also observed in overall microbial community composition (PERMANOVA; df = 1, pseudo-F = 13.84, p = 0.0039) (Fig 3) in repeat sampled TNT dosed lizards over the 14 day exposure period. PERMANOVA further revealed that a relative majority of the variation (45%) in overall microbial community composition could be attributed to the effects of TNT, exceeding the variation attributed by the individual anole.

**Fig 4:**
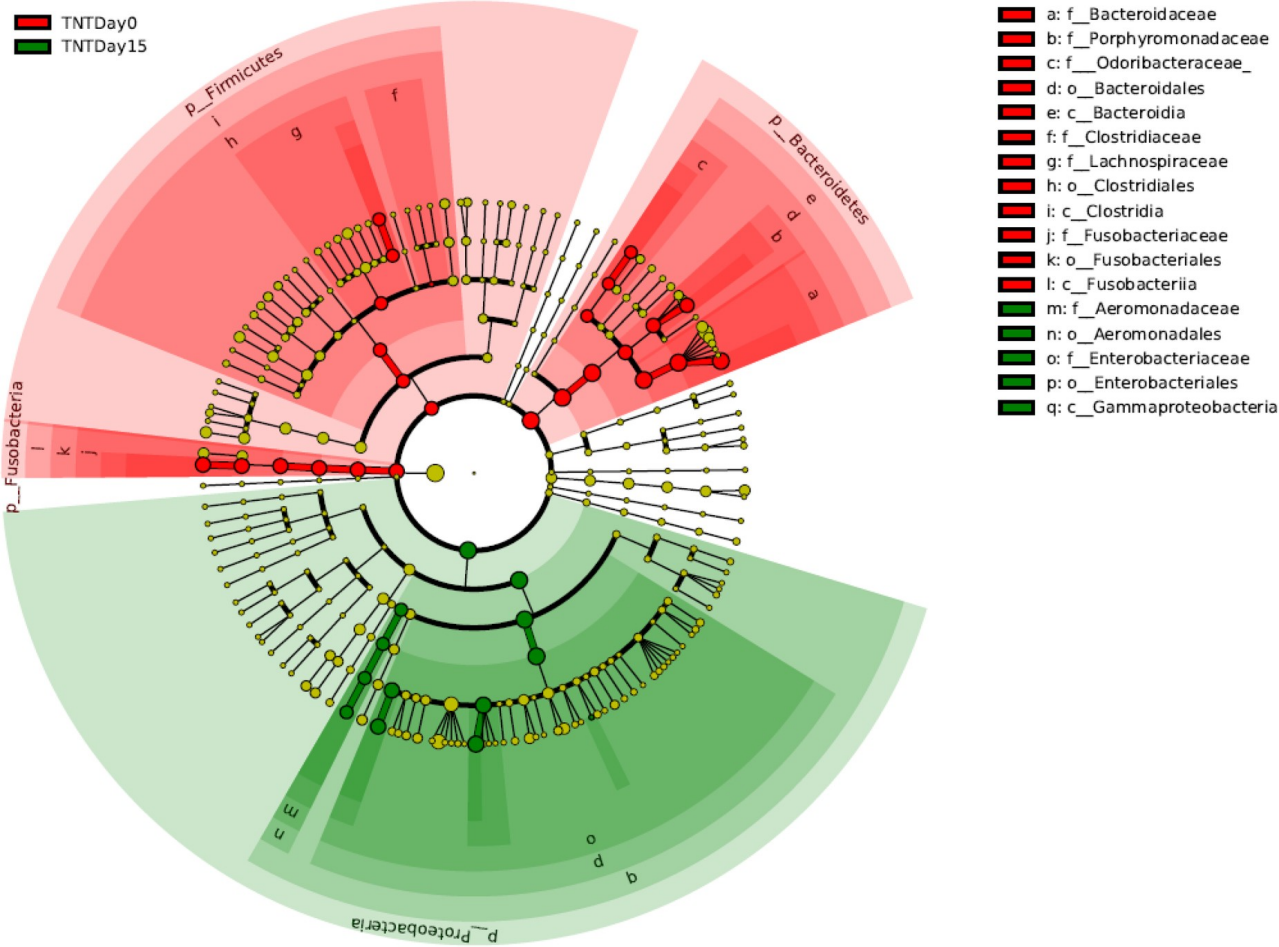
Phylum through family taxonomic cladogram representation of LEfSe analysis of 16S rRNA gene sequences. (Green) Taxonomic groups significantly associated with TNT dosed anoles day 15; (Red) taxonomic groups significantly associated with TNT dosed anoles at day 0. Significance determined using default parameters (Kruskal Wallis test p <0.05 and LDA score >2). The brightness of each dot is proportional to effect size.

**Fig 5:**
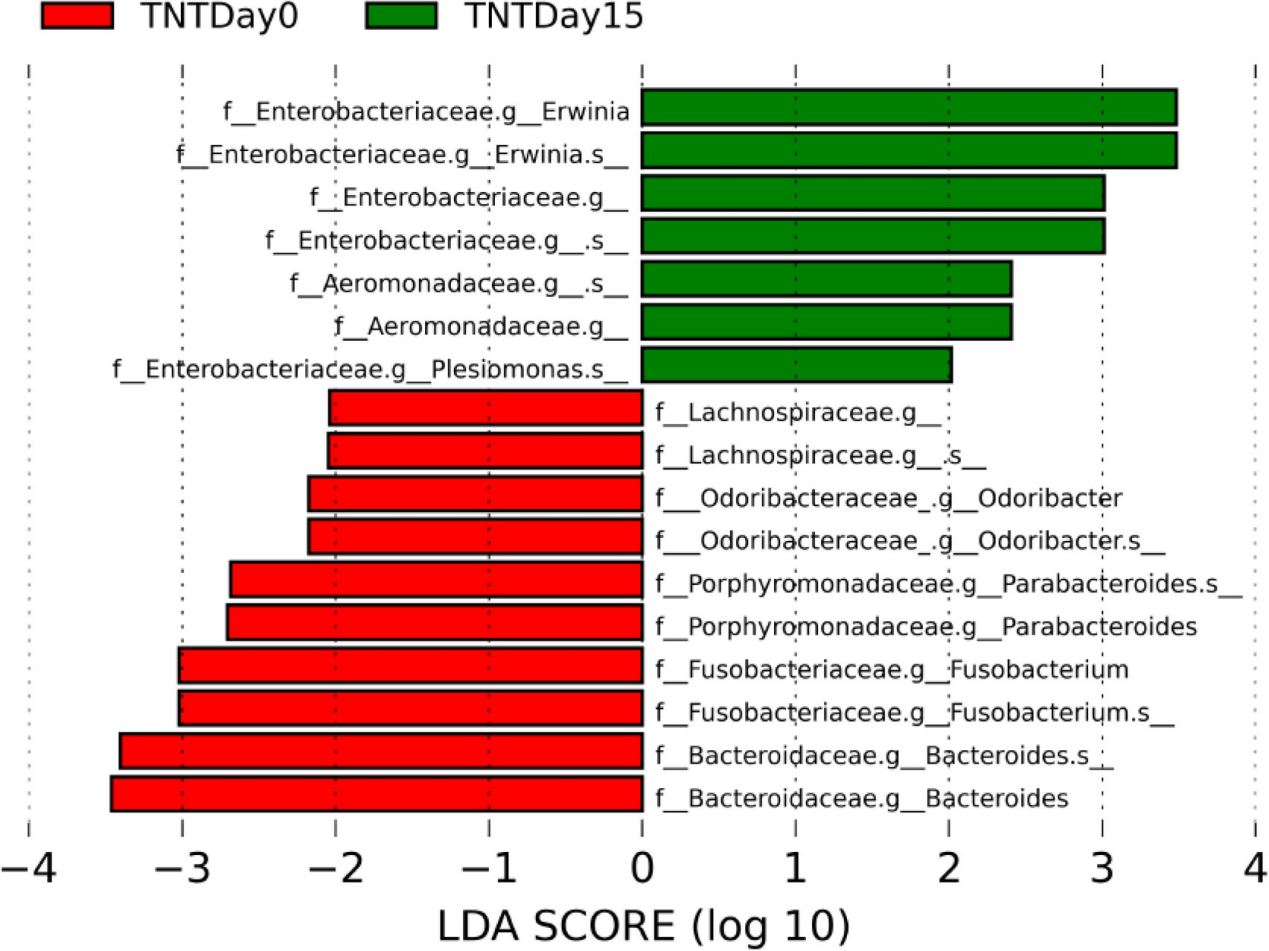
Species level taxonomic histogram representation of LEfSe analysis of 16S rRNA gene sequences. (Green) Taxa significantly associated with TNT dosed anoles at day 15; (Red) taxa significantly associated with TNT dosed anoles at day 0. Significance determined using default parameters (Kruskal Wallis test p <0.05 and LDA score >2). Magnitude of each bar represents the effect size.

An analysis of the relative abundance of community members for TNT dosed anoles revealed that the Enterobacteriaceae were ~ 7-fold higher in the TNT exposed lizards representing 59% of the microbial community compared to only ~ 8% at day 0 prior to dosing (Fig 4) (LDA effect size 3.66, p = 0.0007). Within this family, an operational taxonomic unit (OTU) within the genus *Erwinia* represented 32% of the microbial community in TNT exposed anoles (Fig 5) (LDA effect size 3.48, p = 0.0003).

### Subacute Lead exposure

**Fig 6:**
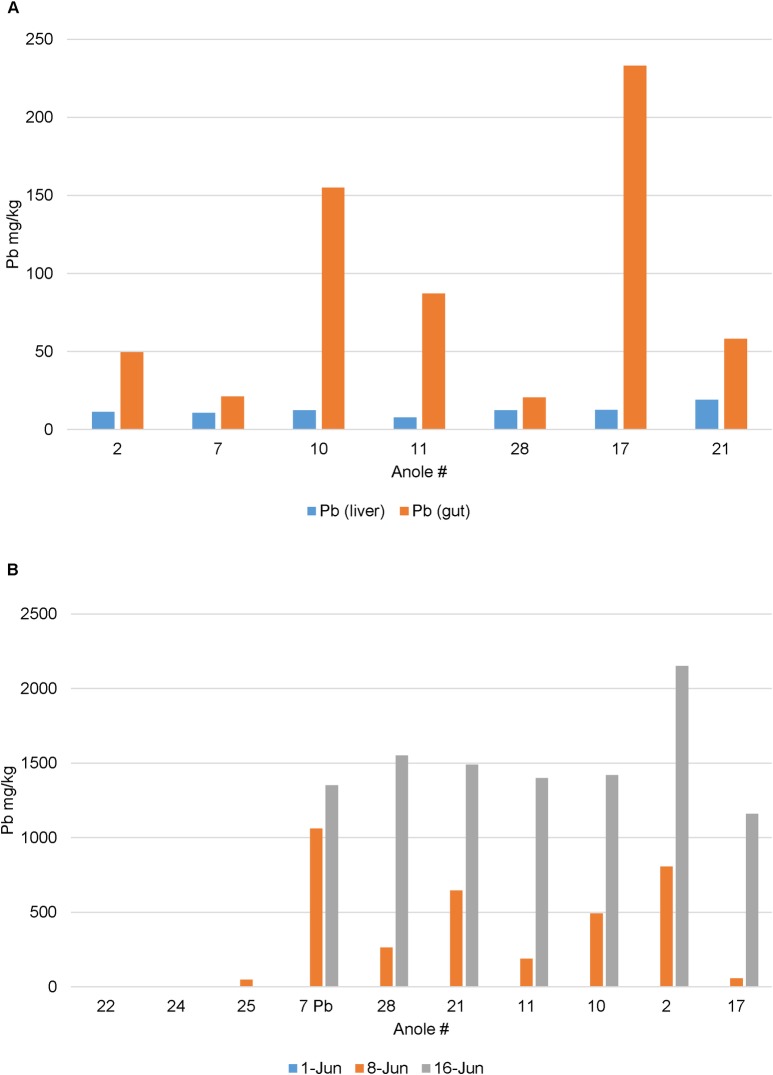
Analysis of lead in liver (A) and fecal (B) samples collected from anoles exposed to lead.

**Fig 7:**
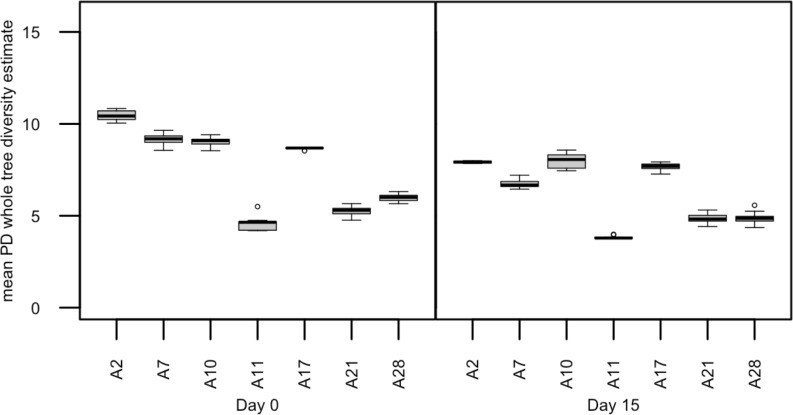
Comparisons of PD whole tree alpha diversity of lead dosed male anoles’ fecal microbiome at day 0 and day 15.

Acclimated male anoles were dosed based on body weight daily with lead nitrate (20 mg/kg) for 14 days. Mean percent weight loss was 0.1% (±9.9%) over the course of the 14-day exposure period suggesting a reproducible, limited lead response to body weight (data not shown). Lead was detected in anole fecal samples as well as in liver tissues (Fig 6). Midpoint (day 7–10) fecal sampling of anoles revealed that lead concentrations increased over the course of the exposure in fecal samples. As a result, microbiome analysis of lead dosed anoles at day 0 and day 15 (n = 7) was performed identical to the TNT dosed animals. Significant differences were observed in PD whole tree diversity (Fig 7) estimates (df = 1, pseudo-F = 18.274, p = 0.0065) but not in overall microbial community composition in lead dosed anoles over the 14 day exposure period (S6 Fig). PERMANOVA further revealed that only ~8% of the variation in the overall microbial community composition could be attributed to the effects of lead.

## Discussion

The overall objective of this research was to establish proof-of-concept that host fecal microbiome composition may serve as a predictive biomarker for exposure to different classes of contaminants. The utility of gut microbiome composition was recently investigated as a pre-clinical tool for identifying exposures to specific heavy metals using the rat animal model and showed some promise towards identifying relevant contaminant transformation pathways [28]. Towards our objective, we used an understudied, ecologically relevant reptile model, the green anole, *Anolis carolinensis* to investigate the effects of the nitroaromatic explosive, TNT, and a metal, lead, on composition of the host fecal microbiome in response to short-term exposures.

Thus far, there has been limited gut/fecal microbiome studies focused on ecologically relevant vertebrate models like lizards [10–16]. Analysis of fecal microbiome samples (n = 20) collected from anoles immediately upon arrival indicated that the majority of the microbial community consisted of the Bacteroidaceae followed the Proteobacteria and Firmicutes. At the family level the relative abundance of lesser microbial taxa were highly variable between individuals. Our community results are similar to what others have reported when examining the effects of ecological and evolutionary diversification on fecal microbial structure in various *Anolis* lizard ectomorphs (trunk-crown, trunk-ground, grass-bush) [16]. Similarly, substantial variations in microbiome composition were observed between and across ectomorphs with the vast majority of organisms characterized as Firmicutes, Proteobacteria and Bacteriodetes. In another study, the role of diet was evaluated in shaping gut microbial communities from the marine iguana (*Amblyrhynchus cristatus*) and land iguanas (*Conolophus sp*.) [10, 11]. Interestingly, at the phylum level, the fecal microbial community in land iguanas was dominated by Firmicutes and Bacteroidetes, similar to what has been observed in anoles. However, marine iguanas, known to consume algae, displayed significantly more *Bacteroides* spp., and members of Lachnospiraceae and Clostridiaceae. And finally a survey of the impacts of diet on the gut microbiome of crocodile lizards revealed the lizards were consistently dominated by Proteobacteria and Bacteriodetes, again emphasizing the importance of these phyla to lizard gut physiology [13].

Due to previously observed variability in relative abundances of microbial taxa that comprise the fecal microbiome in wild-caught anoles [16], anoles were subjected to a two-week acclimation period where they were kept in a controlled environment and fed a consistent diet of crickets. The goal of the acclimation period was to establish a consistent, stable baseline fecal microbiome profile to further elucidate the exposure effects of TNT and lead on microbiome composition. Unfortunately, the two-week acclimation period had no significant effect on microbial alpha diversity or microbial community composition suggesting the acclimation period was too short. However, repeated sampling of each anole allowed for tracking changes in community composition within each individual and each anole was assigned as a random factor in a multivariate statistical model to account for the highly variable bacterial communities associated with wild-caught anoles. As a result, we were able to observe statistically significant reductions in microbial alpha diversity and microbiome composition in TNT dosed anoles. The reduction in microbial alpha diversity of the fecal microbiome in response to a contaminant is consistent with previous studies and indicates that explosives or metals can have a negative impact of members on the bacterial community [28, 37–39]. By contrast, members of the Enterobacteriaceae represented higher proportions of the overall microbial relative abundance in fecal microbiomes of anoles exposed to TNT. At the genus and OTU levels *Erwinia* represented 32% of the microbial community in TNT exposed anoles, a ~25 fold difference from day 0, suggesting this genus could represent a phylogenetic biomarker for TNT exposure. In general, *Erwinia* contains mostly plant pathogenic species and have not been previously identified as part of the anole fecal microbiome [16]. Interestingly, *Erwinia* sp. have been isolated and cultured from crickets and therefore it is possible that this genus was introduced into the anoles via diet and its relative abundance increased in the presence of TNT [40].

Additional functional analysis of the predicted gene content (through LEfSe analysis of KEGG orthologous genes and pathways) of the bacterial communities based on the bacteria identified by 16S rRNA gene amplicon sequencing revealed differences between pre-exposed and TNT-exposed anoles. Among the first level KEGG pathways identified, 170 pathways were found to be significantly associated with pre-exposed (104) and TNT-exposed (66) anoles (S1 Table). Specifically two, first level KEGG pathways encoding for nitrotoluene degradation (KO00633) and toluene degradation (KO00623) were identified that are significantly associated with the TNT-exposed anoles. In addition, three KEGG orthologous genes, a nitroreductase/dihydropteridine reductase, N-ethylmaleimide reductase, and carboxymethylenebutenolidase (K10679, K10680 and K01061) are associated with one of these two pathways and have a role in TNT degradation. Both liver and fecal chemistry data detecting amino dinitrotoluene breakdown products were consistent with TNT reduction possibly resulting from liver and bacterial nitroreductase activity [41–43]. In particular, type I oxygen insensitive nitroreductases have been characterized from a closely related genus, *Enterobacter*, and exhibit nitroreductive activity towards various nitroaromatics [44]. Furthermore, sequenced strains of *Erwinia* encode for predicted nitroreductase containing KEGG pathways providing a possible linkage between the increase in the relative abundance of *Erwinia*, putative nitroreductase activity and the presence of TNT [45].

Studies focused on the transformation potential of explosives and lead by the vertebrate gut microbiome are limited. The sheep rumen is perhaps these best studied system in which the explosives biotransformation potential of rumen fluid and associated bacteria were assessed. Both the explosives TNT and RDX were transformed by either rumen fluid or ruminal microbes under anaerobic conditions [37, 46, 47]. In addition, sheep fed TNT supplements for up to 21 days exhibited a shift in their microbiome composition with an increase in members of the Ruminococcaceae, a response more likely from a ruminant animal system. Similarly, we have demonstrated that termite gut endosymbionts are able to transform the explosives TNT and RDX [48]. While lead, at the concentration used in this study had no effect on the anole microbiome composition, short-term (seven day) studies in zebrafish exposed to 30 μg/L resulted in a reduction in the relative abundance the alpha Proteobacteria and an increase in the relative abundance of Firmicutes [39]. Chronic exposures to lead (0.1 mg/L for 15 weeks) in mice also resulted in variable changes in the relative abundances of Firmicutes and Bacteroidetes [38]. In general, contaminant transformations in the gut are likely the result of anaerobic microbial co-metabolic processes in which the contaminant is fortuitously transformed or adsorbed. Therefore, the selective expansion or reduction of specific microbial taxa in response to a contaminant like TNT may be the result of a bacterial group’s selective ability to metabolize a specific functional chemical group (i.e. reduction of a nitro group to an amino via a bacteria nitroreductase) or in the case of lead, by selectively sequestering or precipitating a metal [49, 50].

The ecological health impacts of environmental contaminants are difficult to predict and as such relevant receptors that are easily interrogated are needed for implementing best management practices at affected sites. Microbiome studies are currently transforming the human health sciences, leading to new paradigms in medicine regarding disease and health. These same insights are relevant to environmental contaminants and their effects on various ecological host receptors and animal models, yet the microbiomes of few ecologically relevant organisms have been described. As a result, the effect of environmental contaminants on microbiome composition and the microbiome’s influence on the transformation of environmental contaminants has largely been ignored in the field of environmental toxicology and risk/hazard assessment. Findings from this study will enhance our fundamental knowledge of the environmental and health impacts of military relevant compounds on terrestrial eco-receptors and their impact on host microbiome composition and metabolism. In addition, changes in microbiome composition, as a result of contaminant exposure, may serve as an easily assayed, noninvasive biomarker for a specific chemical exposure providing enhanced scientific support to risk assessments on military installations [51]. Furthermore, insights from this research could be applied to environmental receptors towards the development of “environmental probiotics” [52]. Such probiotics could offer novel pathways for mitigating some of the biological and toxicological effects caused by exposure to environmental contaminants.

## Supporting information

S1 Fig Comparisons of PD whole tree alpha diversity of wild-caught male anoles’ fecal microbiome at the time of arrival and following 2-weeks of acclimation in captivity. (TIFF)

S2 Fig Principal coordinate analysis (PCoA) based on weighted UniFrac distance among the anole fecal microbiome at the time of arrival and following 2-weeks of acclimation in captivity. (TIF)

S3 Fig Comparisons of the relative abundances representing 1% or greater of the fecal microbial community composition between acclimated and un-acclimated anoles. (TIF)

S4 Fig Principal coordinate analysis (PCoA) based on weighted UniFrac distance among the anole fecal microbiome of control males dosed with soybean oil at day 0 and day 15. (TIF)

S5 Fig Principal coordinate analysis (PCoA) based on weighted UniFrac distance among anole fecal samples and hindgut samples collected at day 0 and day 15, respectively. (TIF)

S6 Fig Comparisons of the relative abundances representing 1% or greater of the fecal microbial community composition between Pb dosed anoles at day 0 and day 15. (TIF)

S1 Table Functional analysis of the predicted gene content (through LEfSe analysis of KEGG orthologous genes and pathways) of the bacterial communities based on the bacteria identified by 16S rRNA gene amplicon sequencing between pre-exposed and TNT-exposed anoles. (XLSX)
