# Supplementary figures and images for: Effects of acute exposures of 2,4,6-trinitrotoluene and inorganic lead on the fecal microbiome of the green anole (Anolis carolinensis)
Source: PLoS One. 2018 Dec 6;13(12):e0208281. doi: 10.1371/journal.pone.0208281 (PMC6283624; doi:10.1371/journal.pone.0208281)

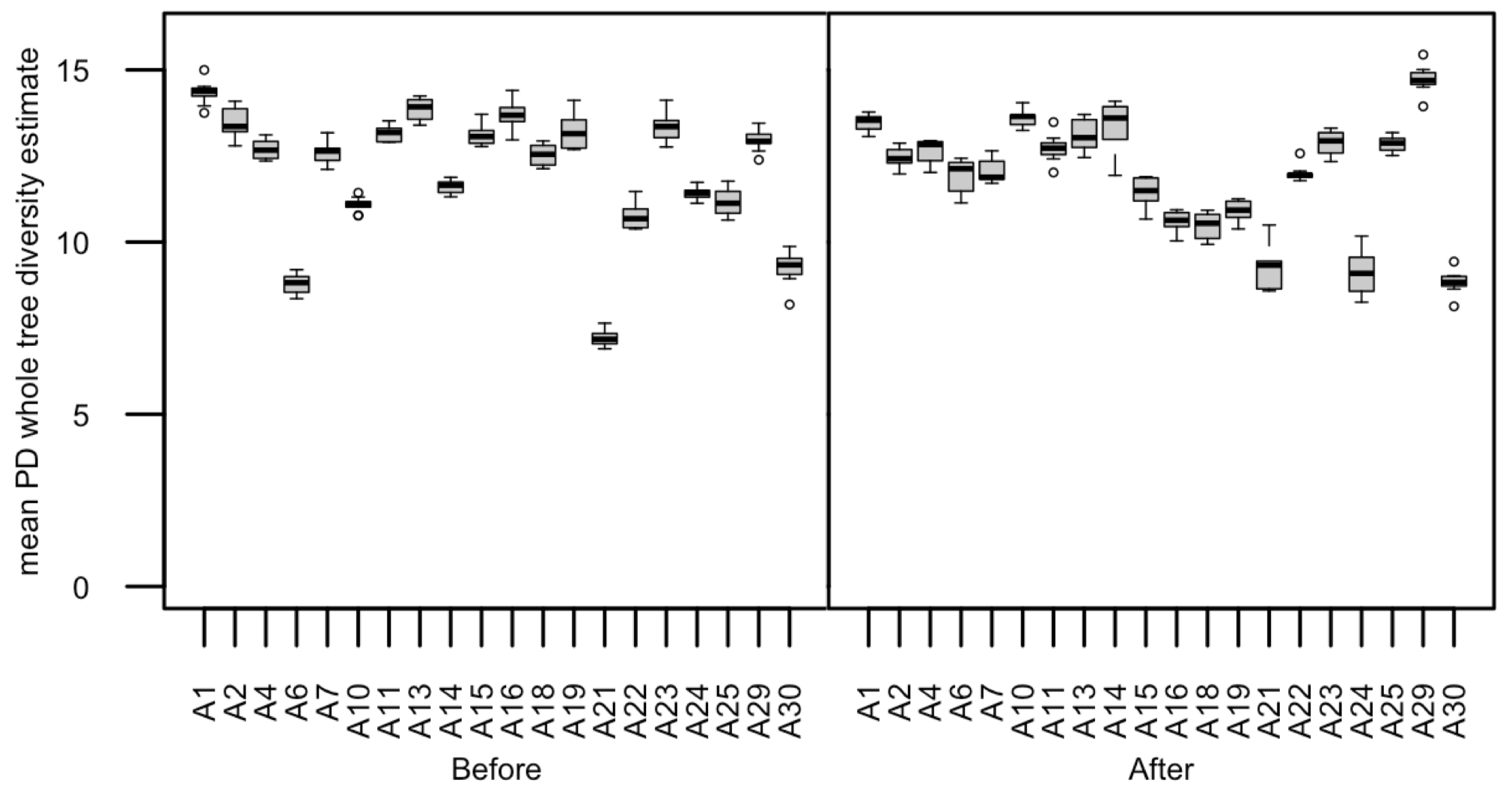

Supplement: S1 Fig — (TIFF) [file pone.0208281.s001.tiff]

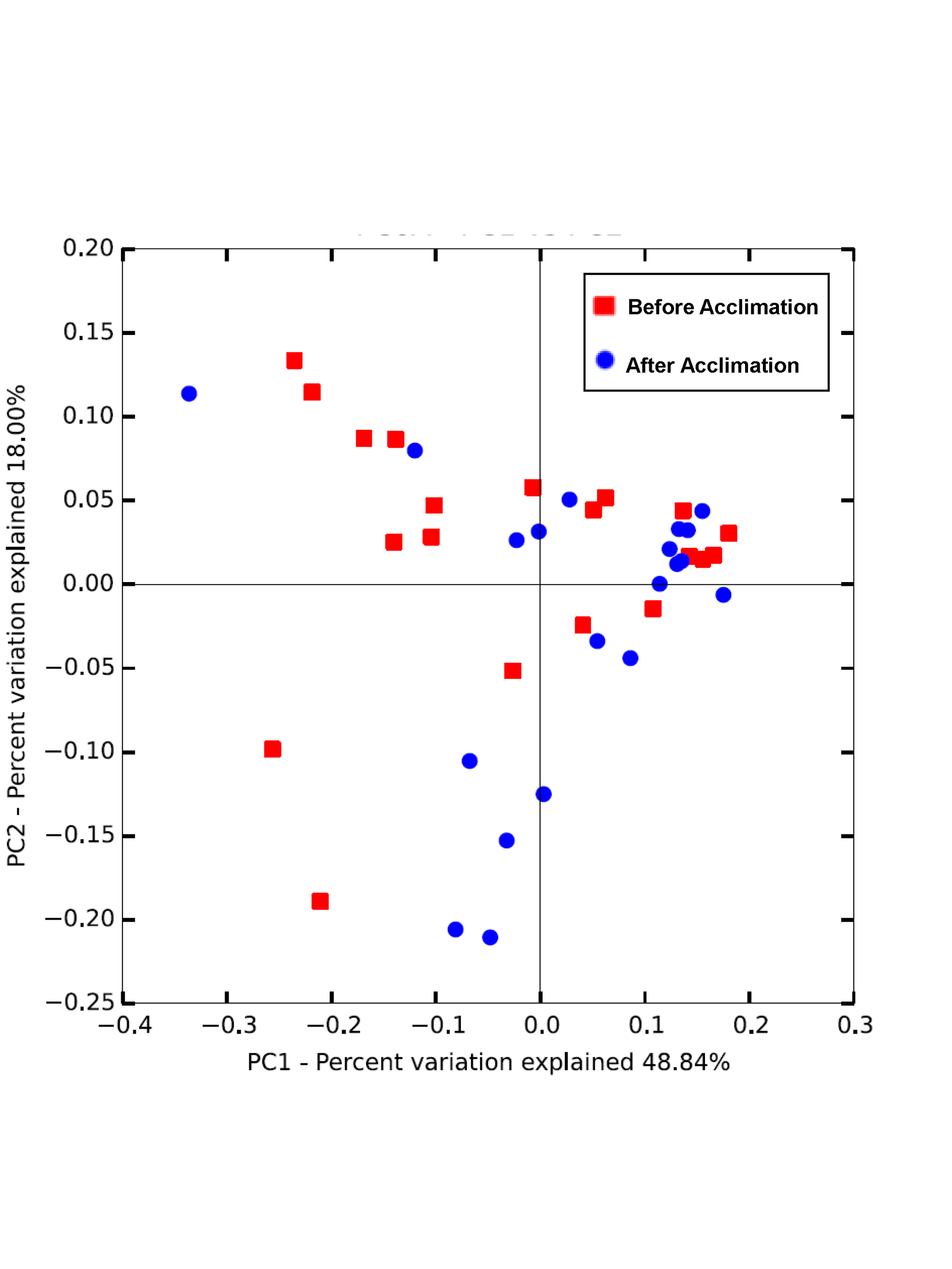

Supplement: S2 Fig — (TIF) [file pone.0208281.s002.tif]

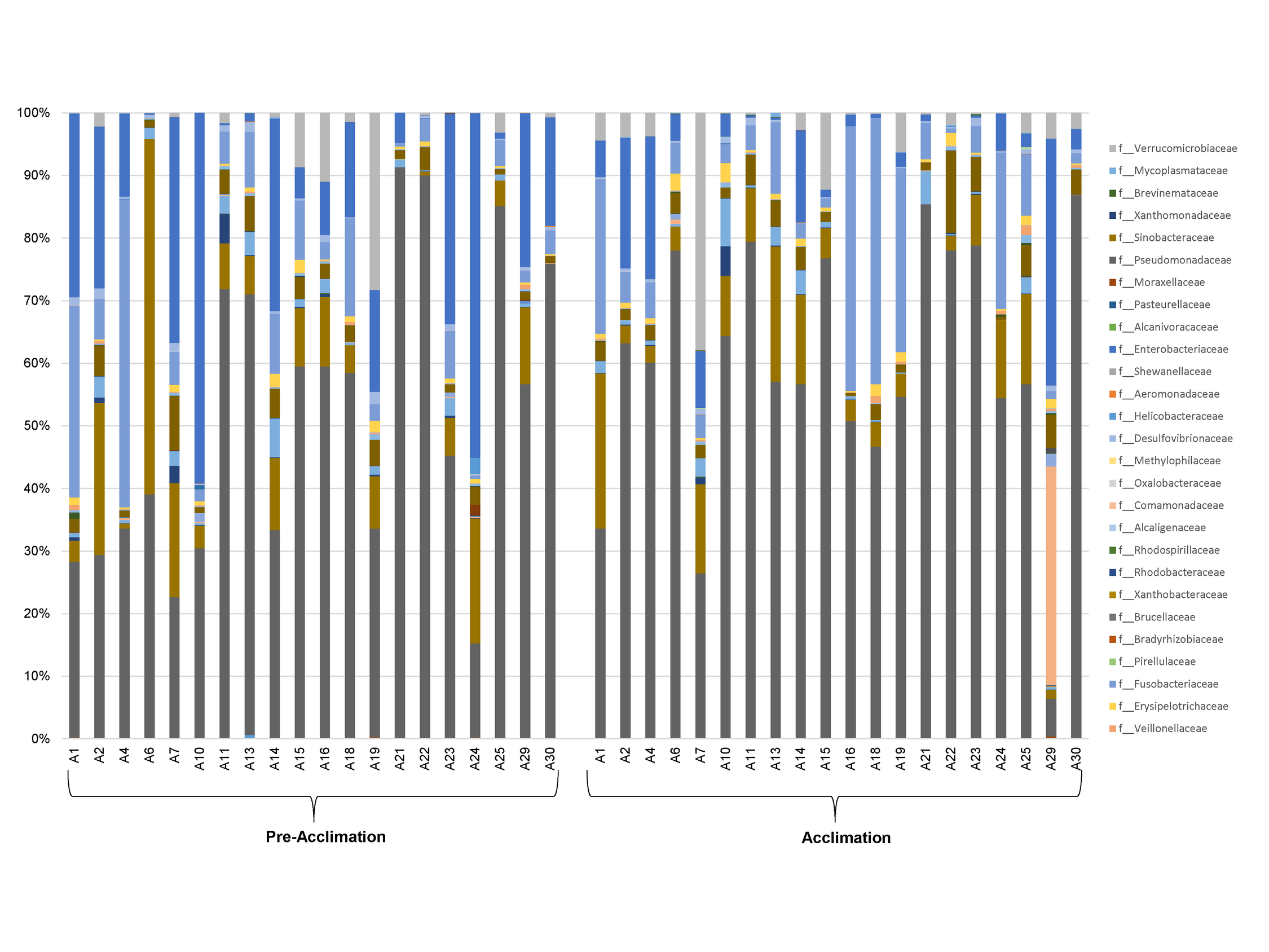

Supplement: S3 Fig — (TIF) [file pone.0208281.s003.tif]

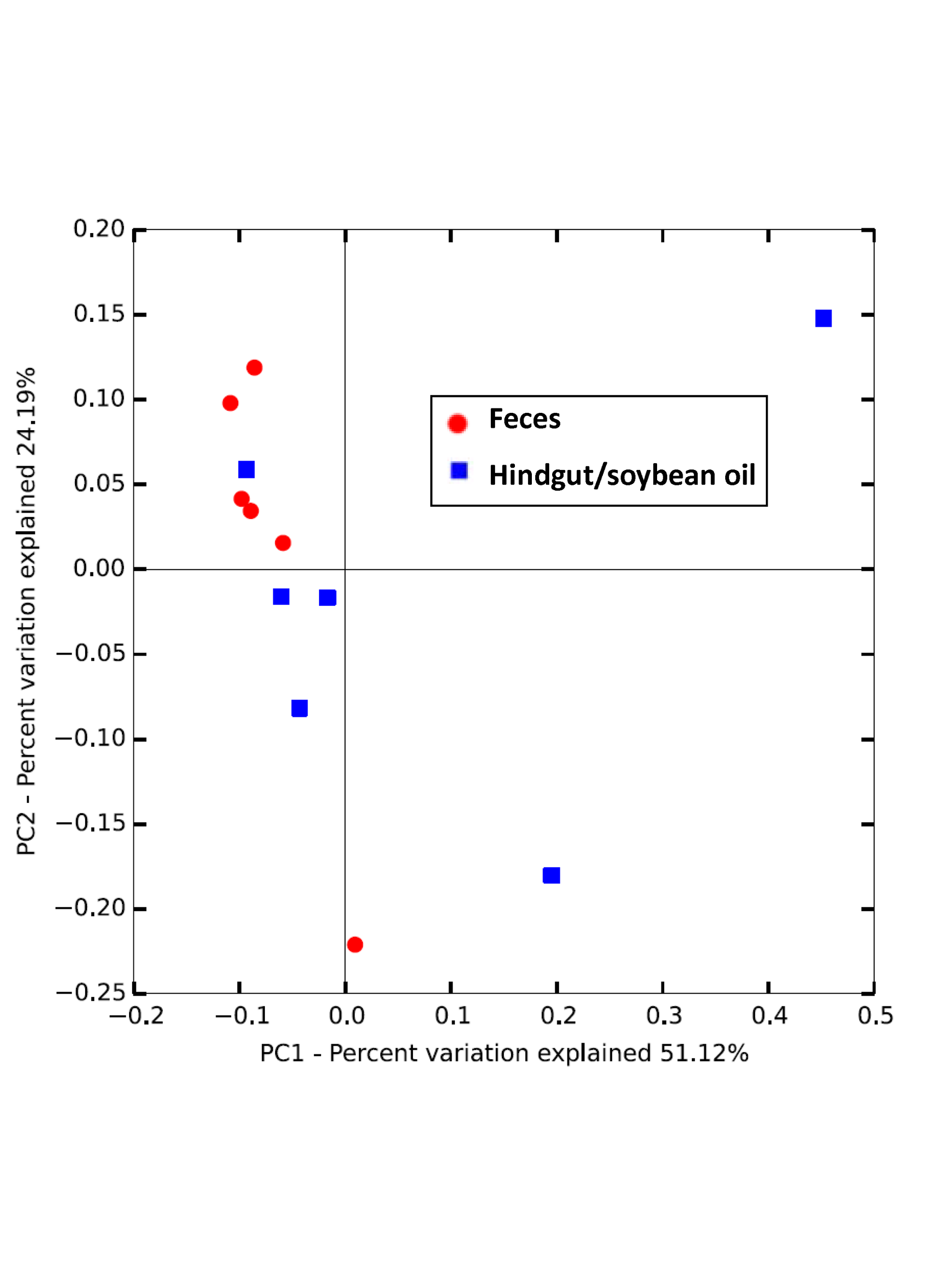

Supplement: S4 Fig — (TIF) [file pone.0208281.s004.tif]

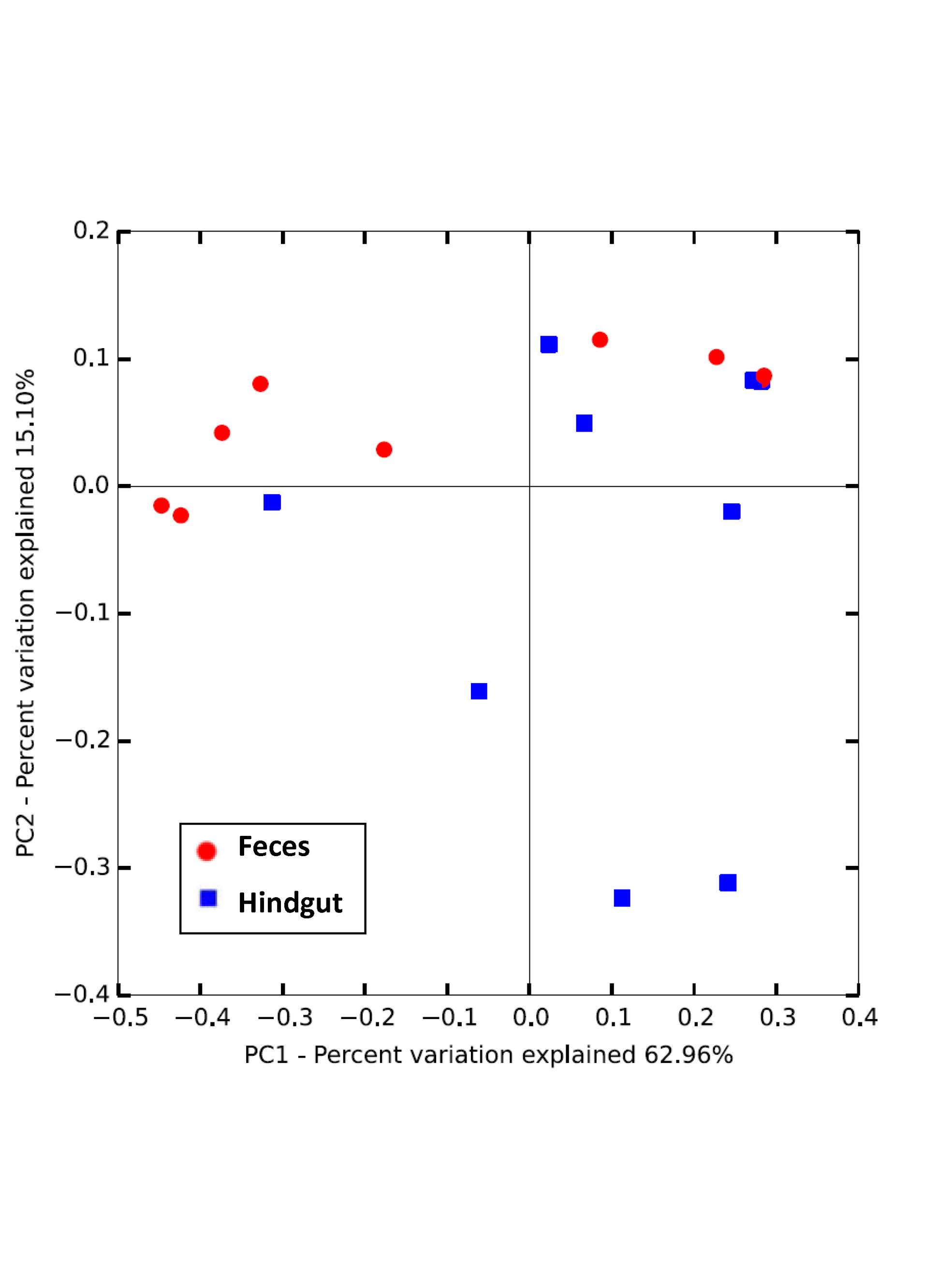

Supplement: S5 Fig — (TIF) [file pone.0208281.s005.tif]

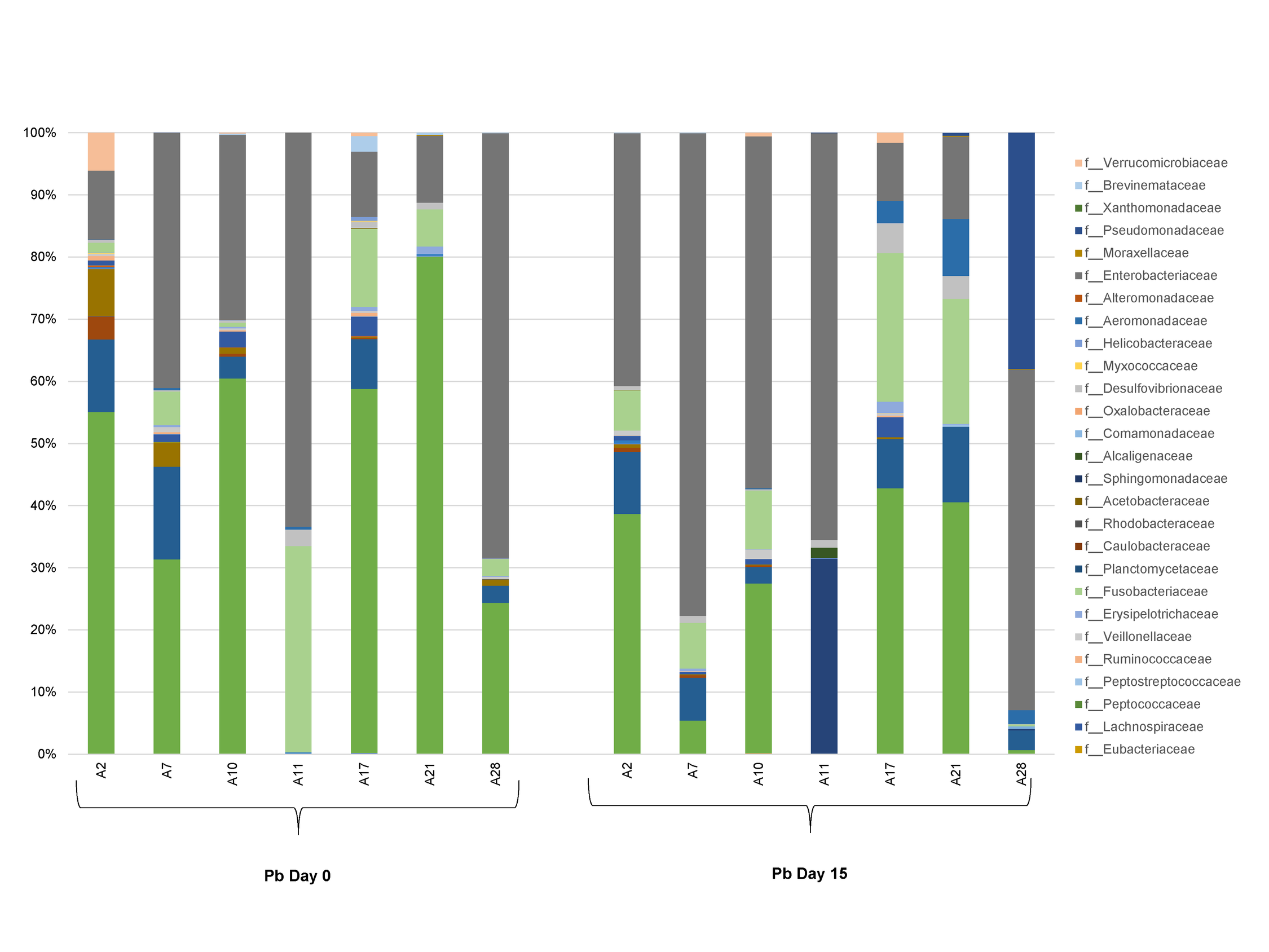

Supplement: S6 Fig — (TIF) [file pone.0208281.s006.tif]
